# Microaggregates as Nutrient Reservoirs for Fungi Drive Natural Regeneration in Larch Plantation Forests

**DOI:** 10.3390/jof11040316

**Published:** 2025-04-16

**Authors:** Yiping Lin, Kefan Wang, Zilu Wang, Xin Fang, Haomin Wang, Nuo Li, Cong Shi, Fuchen Shi

**Affiliations:** 1 College of Life Sciences, Nankai University, Weijin Road 94, Tianjin 300071, China; 2120231449@mail.nankai.edu.cn (Y.L.); kfwang3333@163.com (K.W.); w1012886165@163.com (Z.W.); 1120210521@mail.nankai.edu.cn (X.F.); whmnku@mail.nankai.edu.cn (H.W.); 2120231448@mail.nankai.edu.cn (N.L.); 2 School of Environmental Science and Engineering, Tiangong University, Binshui West Road 399, Tianjin 300387, China

**Keywords:** plantation forest, natural regeneration, soil aggregates, fungal community, co-occurrence network

## Abstract

The natural regeneration of *Larix gmelinii* plantations plays a pivotal role in rehabilitating ecosystem services in Northeast China’s degraded forests. However, mechanistic linkages between soil aggregate nutrient fluxes and fungal community assembly remain poorly constrained. Combining space-for-time substitution with particle-size fractionation and high-throughput sequencing, this study examined successional trajectories across regeneration in Langxiang National Nature Reserve to resolve nutrient–fungal interplay during long-term forest restructuring. The results demonstrated that microaggregates (<0.25 mm) functioned as nutrient protection reservoirs, exhibiting significantly higher total carbon (TC) and nitrogen (TN) contents and greater fungal diversity (*p* < 0.05). Both stand regeneration stage and aggregate size significantly influenced fungal community composition and structural organization (*p* < 0.05). Aggregate-mediated effects predominated in upper soil horizons, where fungal dominance progressively transitioned from Mortierellomycota to Ascomycota with increasing particle size. In contrast, lower soil layers exhibited regeneration-dependent dynamics: Basidiomycota abundance declined with *L. gmelinii* reduction, followed by partial recovery through mycorrhizal reestablishment in *Pinus koraiensis* broadleaf communities. Fungal co-occurrence networks displayed peak complexity during *Juglans mandshurica* germination (Node 50, Edge 345), with 64.6%positive correlations, indicating the critical period for functional synergy. Basidiomycota showed significant negative correlations with nutrients and major fungal phyla (R^2^ = 0.89). This study confirms that natural vegetation regeneration reshapes belowground processes through litter inputs and mycorrhizal symbiosis, while microaggregate management enhances soil carbon sequestration. Near-natural plantation management should incorporate broadleaf species to preserve mycorrhizal diversity and amplify ecosystem services. These findings provide an essential soil ecological theoretical basis for sustainable plantation management in Northeast China.

## 1. Introduction

While industrialization has yielded substantial societal benefits through modern agriculture and industry, it has concurrently accelerated deforestation and land degradation, critically endangering biodiversity and compromising ecosystem services [1,2]. Forest ecosystems serve as pivotal regulators in global carbon cycling, principally through their exceptional carbon sequestration potential [3,4]. Northeast China contains the nation’s largest coniferous forest biome, accounting for one-third of national forest reserves, which is a critical contribution to regional and global carbon neutrality [5]. However, these ecosystems have experienced severe degradation from compounded stressors, including historical anthropogenic disturbances superimposed on natural perturbations like wildfire regimes [6]. Since the early 20th century, large-scale larch plantations have been established to rehabilitate degraded forests and enhance stand structural complexity [7]. As research in ecology progresses, it has become evident that the indiscriminate expansion of forests can have adverse effects on various aspects, including forest species and nutrient cycling [8]. Consequently, under China’s near-natural forest management strategy, addressing biodiversity conservation and ecosystem function optimization in *Larix gmelinii* monocultures has emerged as a paramount scientific challenge [9].

Soil fungal communities, functioning as critical biogeochemical mediators during forest near-natural regeneration, fundamentally regulate forest-soil health through their compositional dynamics and functional plasticity [10,11]. On the one hand, fungi can influence biochemical processes during forest growth by forming symbioses with the root system of forest trees [12]. On the other hand, fungal hyphal networks constitute architectural engineers of soil aggregates, with their spatial configuration and metabolic activities being indispensable for maintaining soil structural integrity and ecosystem functioning [13]. Macroaggregates (>2 mm) predominantly sequester labile organic carbon and harbor arbuscular mycorrhizal fungi, while microaggregates (<0.25 mm) specialize in recalcitrant carbon decomposition mediated by saprotrophic fungal guilds [14]. Microaggregates exhibit significantly greater fungal diversity and abundance under elevated enzymatic activity conditions than macroaggregates [15,16]. Long-term near-natural regeneration of forests may significantly increase the proportion of macroaggregates and enhance the physical protection of soil nutrients by reducing anthropogenic disturbances and promoting the cementation of root secretions and fungal hyphae [17]. Overall, a variety of ecological network relationships, such as mutual competition, parasitism, and predation among fungal groups, determine the fungi’s ability to maintain stability in the face of environmental changes [18,19]. In recent years, fungal network analysis has been widely used in soil studies to identify key taxa and to explore community cooperation or competition [20,21,22]. However, the study of fungal community dynamics and its relationship with soil aggregate nutrients is still in its infancy, especially in the larch plantation forests in Northeast China, where the specific performance and mechanism of action are still unclear. Therefore, exploring the structure and function of soil aggregate nutrients and fungal communities in larch plantation forests under long-term natural regeneration will help us to provide a scientific basis for optimal management, carbon sink function enhancement, and ecosystem service enhancement of larch plantation forests in Northeast China.

As a dominant forest type in Northeast China’s boreal zone, larch plantations exhibit distinct ecological significance. However, the successional dynamics of their natural regeneration processes and associated impacts on aggregate-mediated nutrient fluxes and fungal community assembly remain inadequately characterized. Through longitudinal experimental analysis, this study elucidates the tripartite interactions between soil fungal community dynamics and carbon–nitrogen–phosphorus stoichiometry across aggregate fractions during the natural regeneration of *L. gmelinii* plantations in Northeast China. We posit three mechanistic hypotheses: (1) The natural regeneration of larch forests can significantly change the distribution pattern of soil aggregate nutrients; (2) fungal community assembly demonstrates differential sensitivity to stand developmental stages and aggregate size classes; (3) fungal network architecture exhibits distinct topological configurations across regeneration gradients and aggregate hierarchies. These findings elucidate the mechanistic relationships between forest successional processes and belowground ecological interactions while establishing theoretical foundations for enhancing ecosystem services through sustainable plantation management strategies.

## 2. Materials and Methods

### 2.1. Study Site and Fieldwork Sampling

**Figure 1:**
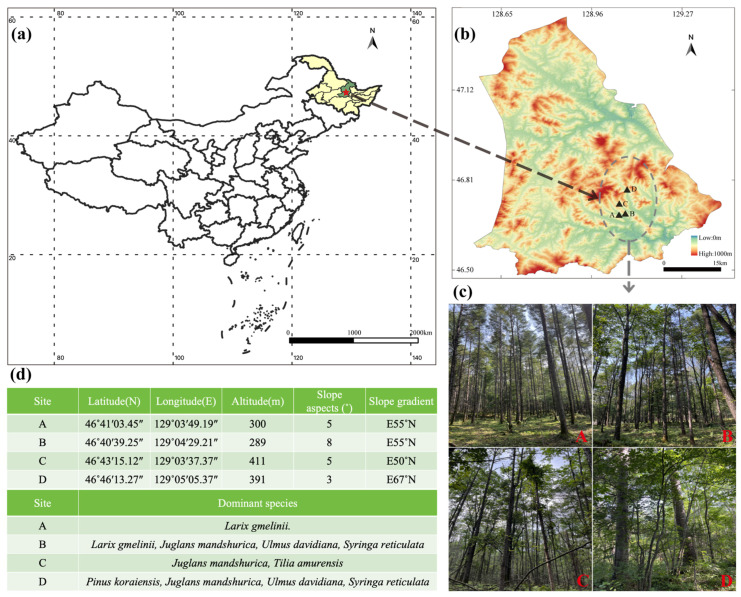
The details of the experimental forest sites and soil sampling. (**a**) Study area location; (**b**) County elevation map (Langxiang National Nature Reserve); (**c**) Field composition of the forest stand; (**d**) Basic information about the forest stand. A, *Larix gmelinii* pure forest; B, *Juglans mandshurica* at the stage of budding; C, *Larix gmelinii* at the stage of mass mortalities; D, broadleaf *Pinus koraiensis*.

The study site is located in Langxiang National Nature Reserve, Heilongjiang Province, China (128°55′30′′–129°15′2′′ E, 46°31′58′′–46°49′38′′ N) (Figure 1b). The site is located in the south-central part of the Xiaoxing’anling Mountains. It is dominated by low hills, with a total area of 31,355 ha, an average elevation of about 610 m, and an average slope of 12°. It has a temperate continental monsoon climate with an average annual temperature of 0.9 °C and an average annual precipitation of 623 mm with dark brown loam as the predominant soil type [6]. Zonal vegetation comprises primary mixed *Pinus koraiensis* broadleaf forests, with characteristic species including *P. koraiensis*, *Juglans mandshurica*, *L. gmelinii*, *Ulmus davidiana, Tilia amurensis* and *Syringa reticulata* [23]. Following extensive *L. gmelinii* monoculture establishment in the 1950s, successional trajectories under combined climate change and anthropogenic pressures have driven ecosystem transition from coniferous dominance to mixed communities featuring *P. koraiensis* and *J. mandshurica* as keystone species.

To assess the long-term regeneration impacts of *L. gmelinii* on soil aggregate nutrient dynamics and fungal community assembly, we employed a space-for-time substitution design, stratifying forest stands into four successional stages based on larch dominance indices. The classification comprises the following: (A) Larch monoculture forest, (B) *L. gmelinii* dominates while *J. mandshurica* emerges gradually, (C) *J. mandshurica* dominates while *L. gmelinii* dies in large numbers and (D) zonal climax community dominated by *P. koraiensis* and *J. mandshurica*, serving as reference ecosystem (Figure 1c). During August 2024, we established three 20 × 20 m replicated plots per forest type (spaced > 100 m), conducting complete vegetation inventories encompassing arboreal, shrub, and herbaceous strata (Figure 1d). Soil sampling followed a five-point protocol: after removing surface organic debris, we collected composite samples at depths of 0–20 cm (topsoil, T) and 20–40 cm (subsoil, S) depths, homogenizing within-plot samples by the horizon. All samples were immediately stored in sterile containers and transported under cryogenic conditions (4 °C).

### 2.2. Soil Aggregate Grouping and Property Determination

Soil aggregates were grouped using the optimum moisture dry sieving method to ensure microbial community stability. The raw soil samples were placed in sterilized aseptic containers and dried at 4 °C until the moisture content was about 15% and then gently crushed through an 8 mm sieve [24,25]. The sieve was then used to divide the soil into three fractions: large macroaggregates (>2 mm), small macroaggregates (0.25–2 mm), and microaggregates (<0.25 mm) [26]. Each soil aggregate fraction was divided into two parts, one for soil nutrient determination, and the other was sent to Guangdong Megger Sequencing Company for fungal identification. Soil pH was determined using the 1:2.5 electrode method [27]. Soil total carbon and total nitrogen were determined by dry burning using a Vario MICRO cube elemental analyzer [28]. Total soil phosphorus was determined using molybdenum antimony UV spectrophotometry [29].

### 2.3. DNA Extraction, PCR, and High-Throughput Sequencing

DNA was extracted from 0.5 g of soil and tested for purity using a kit (Nanodrop One, Thermo Fisher Scientific, Waltham, MA, USA). The nuclear genes of the fungus were amplified by PCR using primers ITS2-2043R (Illumina MiSeq, San Diego, CA, USA). The amplification system consisted of a 50 μL: 25 μM 2×Pre Taq mixture, 10 μM primer F, 10 μM primer R, 50 ng of template DNA, and Nuclease-free water. PCR conditions included pre-denaturation at 94 °C for 5 min, denaturation at 94 °C for 30 s, annealing at 52 °C for 30 s, and extension at 72 °C for 30 s, with 30 rounds of cycling, and 72 °C for 2 min. The products were detected by electrophoresis, and after qualification, the concentration was compared with GeneTools software (ver. 4.03.05.0, SynGene) and the mixed products were confirmed by electrophoresis. Gel recovery (E.Z.N.A. Gel Recovery Kit, Omega, Irving, TX, USA) included eluting the target fragment with TE buffer. Library construction was performed according to the ALFA-SEQ standard procedure, library size was assessed using Qsep400 (BiOptic, Taipei, Taiwan, China), and concentration was detected by Qubit4.0 (Thermo Fisher Scientific, Waltham, MA, USA). Illumina or MGI platform 250 sequencing was performed. Sliding window clipping of reads was performed using fastp v0.14.1, and cutadapt v4.9 removed primers to obtain QC paired-end reads (Clean Reads). USEARCH -fastq_mergepairs was used to filter reads that did not match overlap to obtain raw spliced sequences (Raw Tags). fastp sliding window clipping was used to obtain valid spliced fragments (Clean Tags). USEARCH similarity clustering (97.0%) was used to obtain OTUs, and OTUs were clustered by UPARS to represent sequences with Unite (97.0%). Representative sequences were compared with the Unite (ITS) database to annotate species information.

### 2.4. Statistical Analysis

Differences in soil nutrients, relative abundance, and diversity of fungi among different aggregate fractions or stand types were compared using analysis of variance (ANOVA) in SPSS 26. Significant differences across aggregate size and stand types were visualized using GraphPad Prism 10. Non-metric multidimensional scaling (NMDS) based on Bray–Curtis dissimilarity matrices was conducted using the vegan package in R 4.4.1 to ordinate fungal community structures. Taxonomic composition analysis coupled with linear discriminant analysis effect size (LEfSe) was performed on the Magigene Gene Cloud Platform to identify differentially fungal taxa (http://cloud.magigene.com, accessed on 24 March 2025). Redundancy analysis (RDA) implemented in the Vegan package (v2.6.10) was employed to quantify the constrained variance in fungal communities explained by soil nutrient matrices. Fungal co-occurrence networks were constructed using igraph (v1.5.1) and psych (v2.3.9) packages, with topological parameters calculated to assess network complexity. Network visualization was achieved using Gephi 0.10.1 layout optimization.

## 3. Results

### 3.1. Soil Nutrients and Stoichiometric Ratios

**Figure 2:**
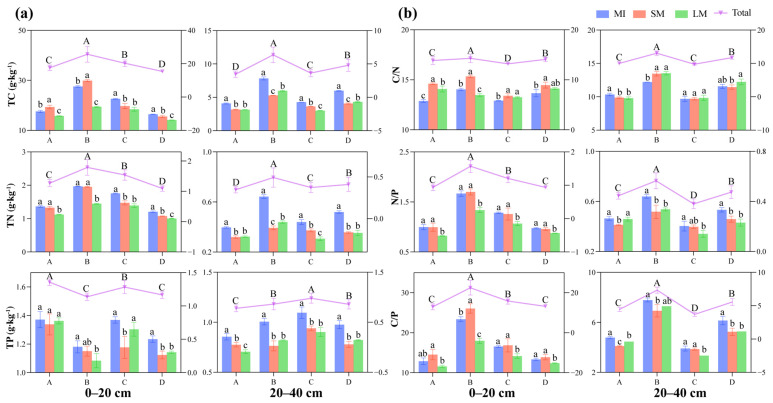
Changes in carbon, nitrogen, and phosphorus whole nutrient fractions (**a**) and stoichiometric ratios (**b**) of different grain size aggregates during long-term forest regeneration. Bars represent means and standard errors of different aggregate fractions for each forest stand expressed in the left coordinate (SEM, *n* = 3). Folded lines represent means and standard errors for each stand expressed in the right coordinate (SEM, *n* = 9). Lowercase letters represent the significance of differences between different aggregate fractions, and uppercase letters represent the significance of differences between stands (*p* < 0.05). MIs, microaggregates; SMs, small macroaggregates; LMs, large macroaggregates. A, *Larix gmelinii* pure forest; B, *Juglans mandshurica* at the stage of budding; C, *Larix gmelinii* at the stage of mass mortalities; D, broadleaf *Pinus koraiensis*.

Long-term forest regeneration processes significantly affected soil aggregate structure and nutrient content (Figure S1 and Tables S1 and S2). All indicators except for the C/N had the highest content in microaggregates, and the nutrient content decreased with increasing grain size. When comparing different natural regeneration stages, all nutrients except phosphorus (TP) had the highest soil nutrient levels at the *J. mandshurica* germination stage (Figure 2a). Specifically, the lowest total nutrient content was found in the topsoil layer of the *P. koraiensis* broadleaf forest and the subsoil layer of the larch pure forest. Regarding stoichiometric ratios, they first increased and then decreased with forest regeneration in the topsoil layer (Figure 2b). In contrast, the lower soil layer had the lowest stoichiometric ratios during the period of mass mortality of larch. Additionally, all nutrients showed significantly higher concentrations in the upper soil than in the lower soil.

### 3.2. Diversity and Abundance of Fungal Communities

**Figure 3:**
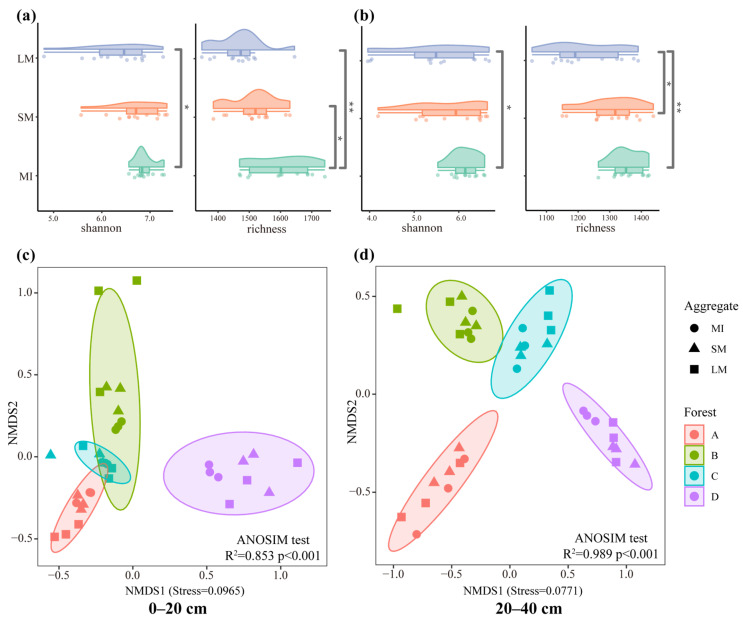
Results of the diversity (**a**,**b**) and NMDS (**c**,**d**) of aggregates of different grain sizes during long-term forest regeneration, where (**a**,**c**) are upper soils (0–20 cm) and (**b**,**d**) are lower soils (20–40 cm). * indicates significant differences (*, *p* < 0.05; **, *p* < 0.01). MIs, microaggregates; SMs, small macroaggregates; LMs, large macroaggregates. A, *Larix gmelinii* pure forest; B, *Juglans mandshurica* at the stage of budding; C, *Larix gmelinii* at the stage of mass mortalities; D, broadleaf *Pinus koraiensis*.

Fungal α-diversity exhibited significant differences (*p* < 0.05) solely between aggregates and between stand types within the subsoil layers (Table S3). Across all soil horizons, the highest α diversity and richness were observed within microaggregates, with diversity decreasing and becoming more erratic as aggregate size increased (Figure 3a,b). Among stand types, the subsoil layer showed the highest diversity and richness during the *J. mandshurica* germination stage and the larch mass mortality stage (Figure S2 and Table S3). NMDS analysis revealed that fungal community structure underwent significant changes during long-term stand renewal (topsoil layer: r^2^ = 0.8534, *p* < 0.01; subsoil layer: r^2^ = 0.9893, *p* < 0.01), with no significant differences among aggregate fractions (Figure 3c,d). Overall, fungal α-diversity showed significant differences between aggregates, peaking in microaggregates, and fungal community structure differed significantly between stands but not between aggregates.

### 3.3. The Community Composition of Fungi

**Figure 4:**
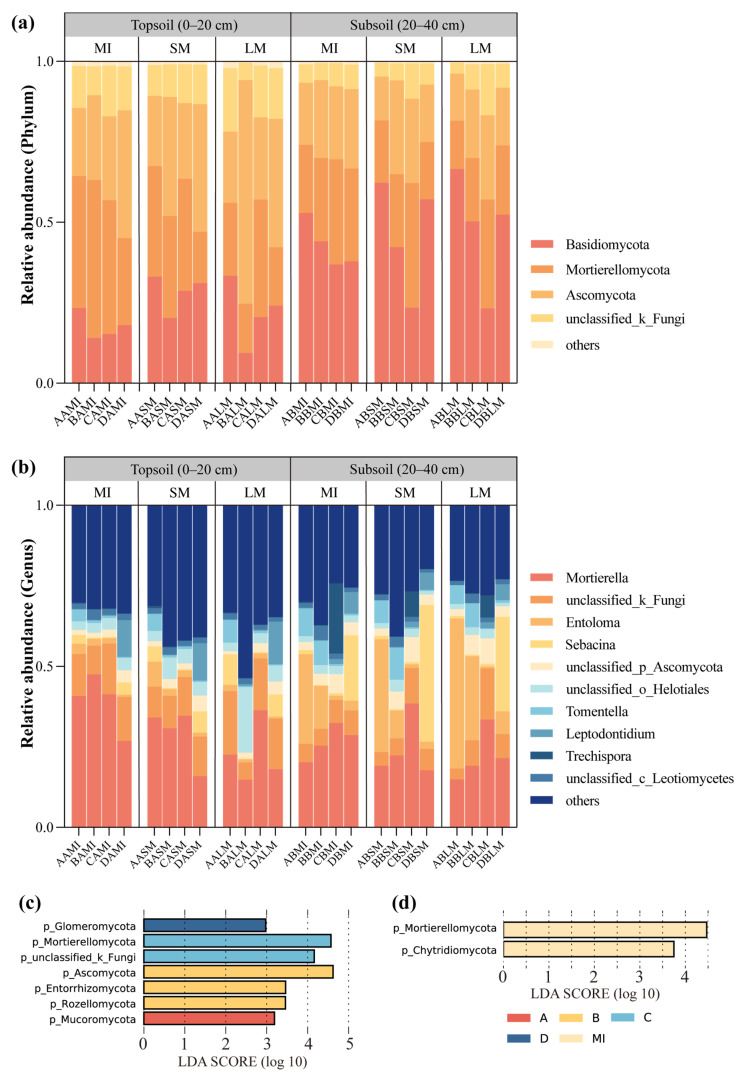
Species richness composition of aggregates of different grain sizes during long-term forest regeneration (**a**,**b**) and linear discriminant analysis (**c**,**d**) at the gate level (LDA > 3), where (**c**) is the result of different stand regeneration stages, and (**d**) is the result between aggregate fractions. MIs, microaggregates; SMs, small macroaggregates; LMs, large macroaggregates. A, *Larix gmelinii* pure forest; B, *Juglans mandshurica* at the stage of budding; C, *Larix gmelinii* the stage of mass mortalities; D, broadleaf *Pinus koraiensis*.

The fungal communities predominantly comprised members of the phyla Basidiomycota, Mortierellomycota, and Ascomycota (Figure 4a). Ascomycota (32.69%) demonstrated peak relative abundance in upper soil horizons, and conversely, Basidiomycota (45.71%) dominated lower horizons (Figure S4). At the genus level, *Mortierella* (27.35%) exhibited the highest abundance across both horizons, while other dominant taxa included *unclassified_k_Fungi* (8.26%), *Sebacina* (5.64%) and *unclassified_o_Helotiales* (3.67%) (Figure 4b and Table S5). Stratigraphic analysis revealed distinct vertical partitioning: Ascomycota and Mortierellomycota dominated topsoil enrichment, whereas Basidiomycota prevailed in lower strata. Mortierellomycota maintained dominance in topsoil microaggregates throughout regeneration stages, showing gradual succession to Ascomycota with decreasing aggregate proportions at larger particle sizes, a pattern that was not obvious in lower horizons (Figure 4a). The composition of fungal communities in the lower soil changed significantly at different stages of stand renewal. The relative abundance of Basidiomycota gradually decreased with stand regeneration, reaching a peak when the stand was transformed into a large number of larch dead, followed by a relative increase in the *P. koraiensis* broadleaf forest. In addition, Mortierellomycota showed the opposite response to Basidiomycota.

**Figure 5:**
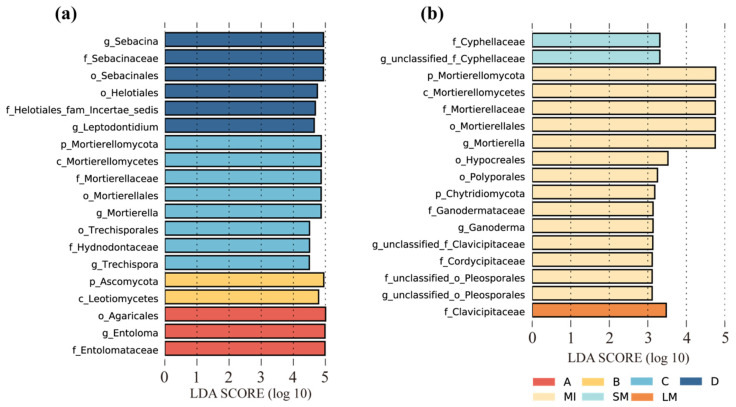
Linear discriminant analyses (**a**,**b**) at the genus level of aggregates of different grain sizes during long-term forest regeneration, where (**a**) is for different stand regeneration stages (LDA > 4.5) and (**b**) is between different aggregate fractions (LDA > 3). MIs, microaggregates; SMs, small macroaggregates; LMs, large macroaggregates. A, *Larix gmelinii* pure forest; B, *Juglans mandshurica* at the stage of budding; C, *Larix gmelinii* the stage of mass mortalities; D, broadleaf *Pinus koraiensis*.

Linear discriminant analysis of effect sizes identified seven biomarkers that served as representative indicators with significantly higher abundance in specific stand types and two with significantly higher abundance in specific aggregate fractions (Figure 4c,d). Mucoromycota was significantly more abundant in pure *L. gmelinii* stands, and Ascomycota, Entorrhizomycota, and Rozellomycota were significantly more abundant in stands with heavy *J. mandshurica* germination. Mortierellomycota showed the highest relative abundance in the stand with heavy mortality of *L. gmelinii*, while Glomeromycota was significantly more abundant in broadleaf *P. koraiensis* forests (Figure 4c). In terms of different aggregate fractions, Mortierellomycota and Chytridiomycota showed significantly higher abundance in microaggregates compared to large macroaggregates (Figure 4d). At the genus level, *Clavicipitaceae* showed higher abundance in large macroaggregates, while *Cyphellaceae* was significantly more abundant in small macroaggregates. *Mortierellaceae* exhibited significant differences in microaggregates (Figure 5 and Figure S4).

### 3.4. The Co-Occurrence Networks of Fungi

**Figure 6:**
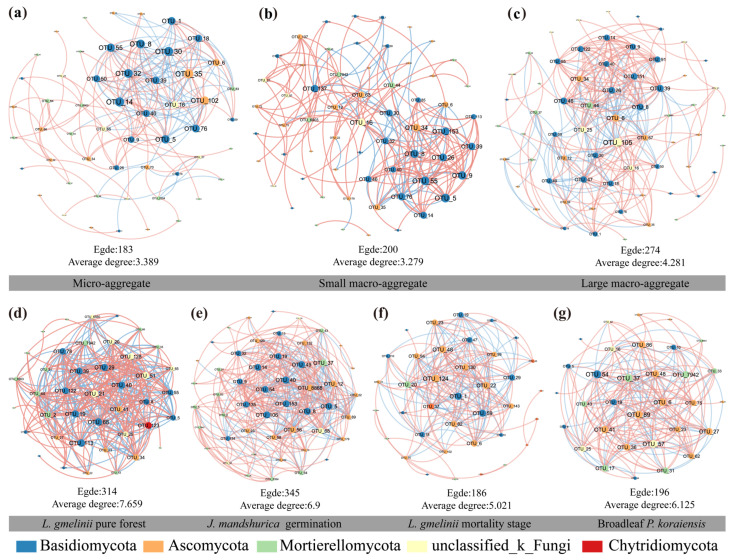
Analysis of microbial co-occurrence networks during natural regeneration of forest stands and in different aggregate grain sizes. (**a**), Microaggregates; (**b**), small macroaggregates; (**c**), large macroaggregates; (**d**), *Larix gmelinii* pure forest; (**e**), *Juglans mandshurica* at the stage of budding; (**f**), *Larix gmelinii* the stage of mass mortalities; (**g**), broadleaf *Pinus koraiensis*.

To explore the symbiotic patterns of fungal communities in different aggregate fractions during the natural regeneration of larch pure stands, we constructed the fungal co-occurrence network by stand type and aggregate fraction (Figure 6). The number of nodes and edges showed fluctuations with stand regeneration, with the highest values recorded during the *J. mandshurica* germination period (50 nodes and 345 edges) (Table S6). The lowest average degree and proportion of positive links occurred during the period of mass *L gmelinii* mortality. And substantially, stand renewal has changed the key taxa dominated by fungi from Basidiomycota to Ascomycota with succession (Figure 6e–g and Table S7). When comparing different aggregate fractions, the point-edge genus increased with increasing aggregate grain size. Additionally, the highest complexity was found in large aggregates (64 nodes and 274 edges), while microaggregates had the simplest structure (54 nodes and 183 edges) (Figure 6a–c). Regarding topological properties, the proportion of positive correlations exceeded 50% in all cases, indicating that cooperation plays a dominant role in the stability of soil fungal communities. The highest levels of cooperation were observed during the *J. mandshurica* germination period (64.64%) and within small macroaggregates (73.50%) (Table S6).

### 3.5. Drivers of Fungal Community Structure

**Figure 7:**
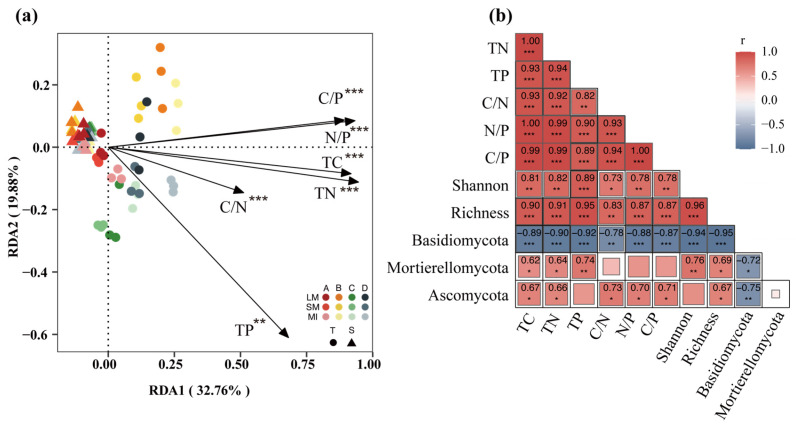
Redundancy analysis of soil nutrients and fungal communities within different aggregate grain sizes during natural regeneration of forest stands (**a**). Correlation analysis between soil total nutrients, stoichiometric ratios, and fungal diversity and dominant clades (**b**). * indicates significant differences (*, *p* < 0.05; **, *p* < 0.01; ***, *p* < 0.001). MIs, microaggregates; SMs, small macroaggregates; LMs, large macroaggregates. A, *Larix gmelinii* pure forest; B, *Juglans mandshurica* at the stage of budding; C, *Larix gmelinii* the stage of mass mortalities; D, broadleaf *Pinus koraiensis*.

Based on the redundancy analysis of soil fungi concerning soil nutrients, the first axis accounted for 32.76% of the structural variation in fungal communities, while the second axis explained 19.89%, with a combined total of 25.65% of the fungal communities’ variation being associated with soil nutrients (Figure 7a). Notably, there were no significant differences in lower soil layers across groups, but upper soil layers showed structural differences between both stand regeneration types and aggregate fractions. Monte Carlo tests revealed that each soil nutrient content had a significant impact on fungal community structure (*p* < 0.05). The relationship between the fungal and soil nutrient characteristics was further investigated using correlation analysis (Figure 7b). The result showed that Basidiomycota had significant negative correlations with all nutrients, but significant positive correlations with Ascomycota except for total phosphorus (TP), while Mortierellomycota exhibited significant positive correlations with all nutrients.

## 4. Discussion

### 4.1. Effects of Regeneration on Nutrients in Aggregates

Forest natural regeneration represents a crucial strategy for transforming the stand structure of pure plantation forests, as stand type and species composition undergo significant changes during long-term regeneration [6]. Soil serves as the foundation for forest growth and development, being influenced by factors such as topography, site conditions, and biological composition [22]. The uptake, utilization, and return of soil nutrients occur throughout each stage of forest natural regeneration. In all aggregate fractions, carbon and nitrogen nutrient contents were high during the *J. mandshurica* germination stage, but total phosphorus (TP) was significantly lower. This may be attributed to the emergence of broadleaf species, which alters the type of litter material and promotes carbon and nitrogen nutrient accumulation. In contrast, phosphorus is less influenced by vegetation and is mainly determined by soil-forming matrices [30]. Broadleaf trees have a greater photosynthetic capacity than coniferous species like larch [31], enabling them to fix more carbon nutrients and return them to the soil through leaf litter. Additionally, coniferous needles contain more lignin and tannins than broadleaf species, leading to slower decomposition and nutrient return rates [32]. This explains the high C/Nat the emergence stage of *J. mandshurica*. The proliferation of broadleaf species and increased photosynthetic rates have enhanced carbon nutrient levels and led to substantial nitrogen uptake, resulting in higher nitrogen deficiencies and C/N. The low upper soil nutrient content in broadleaf *P. koraiensis* forests contrasts with most studies. This discrepancy may be due to the richer species diversity of litter material after long-term natural forest regeneration, which enhances soil water eluviation. However, elevated water content also increases leaching, causing nutrients to be stored in deeper soil layers [33]. Chemosensitization between *L. gmelinii* and *J. mandshurica* mitigates the autotoxicity of *J. mandshurica* and promotes its rapid growth at the emergence stage [34]. Overall, the presence of broadleaf species such as *J. mandshurica* has increased nutrient content in pure larch forests but has also led to the problem of microbial–plant nitrogen competition.

Soil aggregates, as a basic component of soil structure, are formed mainly by mineral particles under the combined influence of abiotic factors such as organic matter and biotic factors such as microorganisms [35]. Soil aggregates act as the main carriers of organic matter, encapsulating carbon, nitrogen, and phosphorus nutrients to form protection, thereby maintaining soil fertility [36]. Generally, soil carbon and nitrogen nutrients often decrease with increasing particle size of aggregates, but there is no clear conclusion at this stage [37]. In our study, nutrient content increased with decreasing particle size. This pattern may be due to less disturbance of microaggregates during long-term natural regeneration. As a result, microaggregates formed a better protective association with nutrients, which is consistent with the results of Zhang [38,39]. Moreover, microaggregates have a higher specific surface area than macroaggregates, making them more likely to adsorb organic matter [40,41]. The lower C/N ratio of microaggregates supports this hypothesis. This low ratio suggests that microaggregates have sufficient nitrogen nutrients, which are more conducive to enzyme renewal and the development of microbial components to promote carbon and nitrogen mineralization. Stoichiometric ratios responded differently to aggregate alteration in upper and lower soils. Upper soils had higher ratios in small macroaggregates, while microaggregates had higher ratios in lower soils. Higher C/P and N/P ratios indicate reduced phosphorus effectiveness, with the highest phosphorus effectiveness found in large macroaggregates [42]. This may be because phosphorus is significantly affected by soil composition. Macroaggregates, more likely to contain sand and gravel, can be broken down into usable phosphorus by enzymes and microorganisms [30]. As soil depth increases, the influence of soil lithology intensifies, reducing the impact of litter material. Consequently, phosphorus limitation shifts from small macroaggregates to microaggregates.

### 4.2. Effects of Forest Regeneration and Aggregate on Fungal Community Diversity and Structure

Early studies of forest vegetation succession sequences in the Xiaoxing’anling have provided an adequate vector base for soil microbial exploration in boreal coniferous forests [6]. Forest stand-type changes can influence soil nutrients through litter inputs and root secretions, which can alter fungal composition and structure [43]. It has been found that the abundance of fungi increases with forest succession, but the diversity decreases somewhat at later stages, which is related to the soil nutrients required by the microbial community [44]. In this study, soil fungi did not show significant differences in different periods of forest stand renewal. This may be since fungi, unlike bacteria, are less affected by plant diversity and are mainly influenced by litter changes that affect fungal diversity [45]. Additionally, fungi are resistant to environmental changes compared to bacteria [46], which may also explain the lack of significant differences in their diversity [47]. Aggregate fractions have a stronger effect on fungal diversity than stand renewal. Aggregates with different particle sizes significantly affected fungal diversity due to their unique physicochemical properties [48], mainly increasing diversity with decreasing particle size. This may be due to the distribution of nutrients in the soil as most of the organic matter is aggregated in microaggregates, nourishing more fungal populations [49]. The findings of our study are consistent with those of previous research, which concluded that taxonomic richness is higher in microaggregates [16].

The results of the NMDS analyses reveal a significant shift in the structure of the fungal community at different stages of planted forest stand regeneration, consistent with previous research findings [50]. Soil fungal communities are mainly influenced by the root system of forest trees during the formation process and form a close trophic relationship with plants [51]. Vegetation turnover during stand regeneration, such as the mass germination of *J. mandshurica* or mass mortality of *L. gmelinii*, alters the proportion of organic nutrient storage in the soil, ultimately leading to changes in the structure of the fungal community. However, the structure of fungal communities was not significantly affected by aggregates, likely due to the aggregate formation process [52]. Microaggregates gradually form macroaggregates through the binding action of fungal hyphae and other substances [53]. Fungal hyphae interact among aggregates, resulting in a fundamentally uniform composition.

### 4.3. Forest Stand Renewal and Dominant Fungal Taxa in Aggregate Fractions

Long-term forest regeneration induces changes in fungal community composition, leading to significant differences across various soil layers [54]. Basidiomycota, a widely distributed fungal phylum in temperate and coniferous forests, often form mycorrhizal associations with hosts in the inter-root zone to boost root system growth and development [55]. With increasing soil depth, root system density changes, consequently altering fungal community composition. Mortierellomycota, as saprophytic fungi, usually colonize the humus layer and play a dominant role in the decomposition of recalcitrant organic matter [56]. In contrast, Ascomycota excels at decomposing cellulose-and lignin-rich apoplasts [57]. Microaggregates can effectively encapsulate recalcitrant organic carbon due to their small size [58]. Cellulose macromolecules formed from litter debris increase with aggregate particle size [59], which may explain the shift from Mortierellomycota to Ascomycota as aggregate size increases. Additionally, large macroaggregates require more roots and exudates for cementation than microaggregates [60]. This is supported by LDA results showing *Clavicipitaceae* composition changes in macroaggregates. The *Clavicipitaceae* family includes several symbiotic fungi capable of facilitating the formation of large aggregates through mycelial entanglement, which includes many symbiotic fungi capable of facilitating the formation of large macroaggregates through mycelial entanglement [61]. Based on fungal aggregation in different fractions, *Cyphellaceae* in small aggregates degrade macromolecules like lignocellulose [62], and then *Mortierellaceae* in microaggregates further break down residual chitin or lipids [63].

During the exploration of stand regeneration, it was observed that the abundance of Basidiomycota initially decreased and then increased with stand development, being the lowest in *J. mandshurica*-dominated stands. This might result from Basidiomycota’s common symbiotic relationships with Pinaceae roots to drive population renewal and succession [64]. Some of these fungi in Basidiomycota, such as *Suillus grevillei*, are highly specific to larch, while others like *Amanitaceae* can form mycorrhizae with *P. koraiensis*, enabling nutrient sharing with other broadleaf trees [65]. Consequently, their content was higher in early larch pure stands and gradually decreased as larch and other species declined, with the eventual emergence of red pine in the top community. Linear discriminant analysis revealed a gradual shift in the dominant fungal phylum during forest stand natural regeneration. The process started with Mucoromycota, which decomposes easily degradable carbon sources, and then transitioned to Ascomycota, which breaks down difficult to degrade organic matter, and Mortierellomycota, which decomposes lipids [63], and this shift gradually formed a carbon cycle network. The main component of Glomeromycota, arbuscular mycorrhizae, expands plant root ranges through mycelial networks and helps form soil aggregates [66]. Thus, Glomeromycota is used as a key discriminator in the broadleaf red pine forest stage, which affects microbial activity by transporting nutrients via mycelia and improves soil structure by promoting aggregate formation.

Network-based analysis methods reveal potential interactions between fungal communities and elucidate microbial species association complexities through topological network structures [67]. Theoretically, higher positive-to-negative linkage ratios and increased modularity suggest greater stability and adaptability in fungal communities [24]. In our study, during regeneration, larch pure stands significantly impacted fungal community stability. With the emergence of broadleaf species like *J. mandshurica*, fungal diversity increased. At this point, the fungal community exhibited the highest number of point edges and positive correlations in the network, which elevated community complexity indicated a stable survival state [68]. However, over time as larch died, the number of point edges, average degree, and positive correlation ratio of key fungal colonies substantially decreased, reducing fungal stability [69]. These findings strongly indicate that changes in soil fungi can predict trends in plant taxa. Analysis of the fungal mutualistic networks in soil aggregates of varying particle sizes revealed that small aggregates exhibited the highest proportion of positive fungal mutualistic relationships. This suggests that their fungal communities achieve greater structural stability through synergistic metabolic interactions, potentially enhancing functional redundancy during microenvironmental fluctuations. In contrast, larger aggregates displayed significantly higher mean network connectivity, indicating a network architecture that buffers external disturbances via redundant pathways. This aligns with the ecological role of large aggregates as resistant units in soil systems. Collectively, these findings indicate that optimizing the size distribution of soil aggregates not only enhances soil hydro-meteorological conditions but also modulates fungal mutualistic patterns, which in turn indirectly supports plant growth and development.

### 4.4. Interactive Effects of Forest Regeneration on Nutrient and Fungal Communities Within Different Aggregate Fractions

Soil total carbon and total nitrogen, key organic matter components and soil nutrient indicators, are primary drivers of soil fungal diversity (Figure S3) [70,71], consistent with Zhang’s findings that soil nutrients influence fungal community structure [72]. They serve as the main nutrient supply substrate, impacting fungal community composition and metabolic activities. Basidiomycota showed highly significant negative correlations with all measured components. This may be because Basidiomycota decomposes lignin to release carbon, nitrogen, and phosphorus nutrients in the mineral-bound state [73]. However, during the rainy season, increased soil water content leads to rapid nutrient absorption by plants or leaching from the soil, preventing long-term soil retention. In our study, Basidiomycota exhibited significant changes in relation to forest stand development. This pattern is likely due to shifts in aggregate proportion. During the early stages of stand regeneration, small macroaggregates predominated, with *Mortierellaceae* being the dominant fungal group. In contrast, during later stages, the proportion of microaggregates increased, allowing *Cyphellaceae* to become more prominent. These compositional shifts intensified resource competition among soil fungal communities, which may explain the high proportion of negative correlations observed in the microbial network during the late stages of larch mortality. Additionally, Basidiomycota can dominate in infertile soils through efficient organic matter decomposition, suppressing simple carbon source fungi in Ascomycota [74]. Its secondary metabolites, containing terpenoids or phenolic acids that inhibit spore germination or mycelial growth, reduce fungal diversity [75]. This is supported by the chemosensitization between *L. gmelinii* and *J. mandshurica*, where terpenoids or phenolic acids were found in larch root extracts. Overall, natural forest regeneration and vegetation changes largely determine the subsurface ecosystem environment, influencing the structure of fungal communities. Species composition is one factor affecting soil properties and microbial activity, but microhabitat influences like cluster structures should also be considered in specific analyses.

One limitation of this study is that the absence of active nutrient indicators or enzyme activity profiles represents a potential gap in our understanding of soil fungal community dynamics. These factors are likely to play a significant role in shaping fungal community composition and will be a key direction for future exploration. Furthermore, this study only focused on changes in fungal communities during forest stand renewal in Northeast China. However, future research should broaden its scope to explore how soil aggregation dynamics influence fungal community assembly during forest restoration, particularly in degraded ecosystems across Asia and globally. This expanded perspective will provide a more comprehensive understanding of the ecological factors shaping fungal diversity and function in recovering forest systems. In the future, the extent to which vegetation succession and regeneration contribute to global forest ecosystem services can be investigated by incorporating appropriate aggregate fraction information into the ‘plant–soil-fungi’ intersystem. Given global climate change, appropriate forest management measures can be developed to improve soil ecosystem functioning through soil microbial changes, achieving effective long-term contributions to nutrient cycling and carbon fixation.

## 5. Conclusions

This study utilized the natural regeneration process of *L. gmelinii* plantations in Langxiang National Nature Reserve to examine soil aggregate nutrient dynamics and fungal community restructuring during vegetation succession. Forest regeneration altered aggregate-scale nutrient stoichiometry. *J. mandshurica* establishment enhanced microaggregate C and N contents, while P availability remained constrained by geochemical factors. Fungal assemblages demonstrated sensitivity to both successional stage and aggregate size classes, with decomposition processes exhibiting particle-size-dependent gradients. Network topology analysis revealed successional impacts on fungal interactions, in which cooperative dominance establishment shifted to competitive regimes in senescent larch stands. These dynamics emerged from the interplay of biodiversity shifts, nutrient reallocation, and silvicultural interventions. The result of this study advances our understanding of microhabitat-scale fungal ecology and provides mechanistic insights for the adaptive management of plantation ecosystems under global change.

## Data Availability

The original contributions presented in this study are included in the article/Supplementary Material. Further inquiries can be directed to the corresponding author.

## Supplementary Materials

The following supporting information can be downloaded at https://www.mdpi.com/article/10.3390/jof11040316/s1: Figure S1: Soil aggregate fractions under long-term neutral regeneration. Figure S2: Fungal diversity during natural regeneration stages in different stands. Figure S3: Random forest analysis of soil nutrient and fungal diversity within different aggregate grain sizes during natural regeneration of forest stands. Table S1: Nutrient and stoichiometric ratios within soil aggregate fractions at different stages of stand regeneration. Table S2: Two-way ANOVA analysis of soil nutrition. Table S3: Alpha diversity of fungal communities. Table S4: Relative abundance (%) of fungal phyla (top 5). Table S5: Relative abundance (%) of fungal genera (top 10). Table S6: Fungal covariance network topology of forest stand renewal processes and aggregate components. Table S7: Key taxa based on covariate network results for different stand natural regeneration stages. Table S8: Key taxa for different agglomerate fractions based on covariance network results.
