# Supplementary material for: Microaggregates as Nutrient Reservoirs for Fungi Drive Natural Regeneration in Larch Plantation Forests
Source: J Fungi (Basel). 2025 Apr 16;11(4):316. doi: 10.3390/jof11040316 (PMC12028414; doi:10.3390/jof11040316)
Supplement: Supplementary file 1 [file jof-11-00316-s001.zip › jof-3576313-supplementary.pdf]

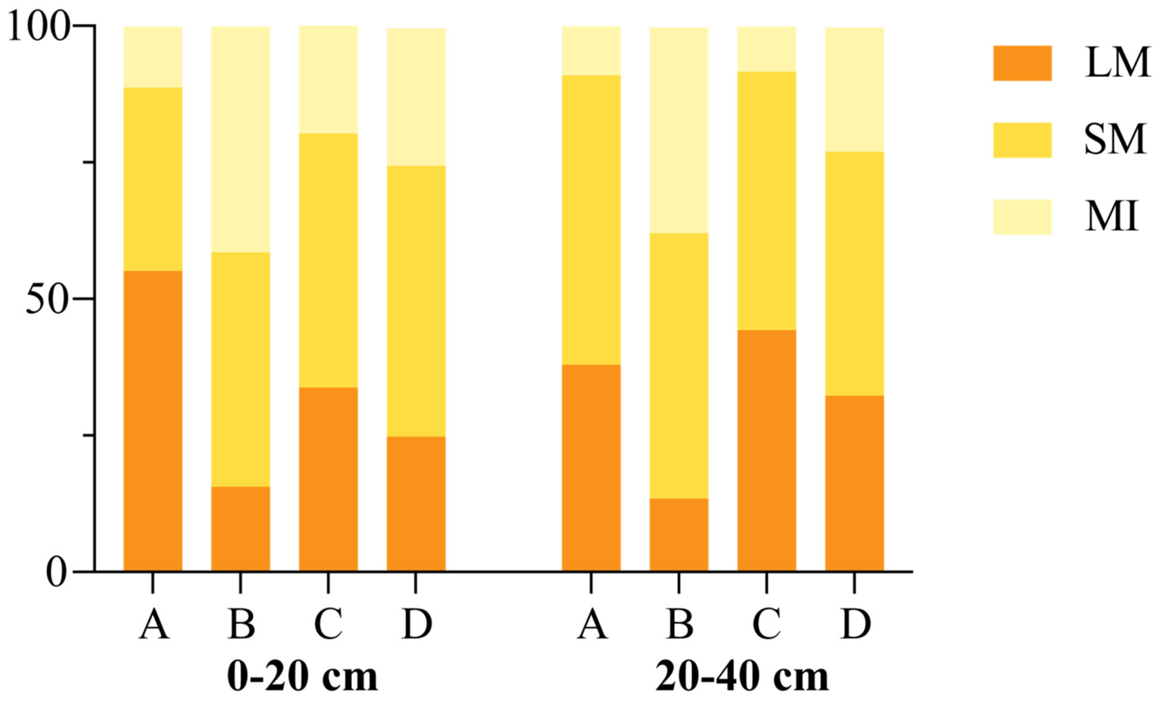

Figure S1. Soil aggregate fractions under long-term neutral regeneration. A, *Larix gmelinii* pure forest; B, *Juglans mandshurica* at the stage of budding; C, *Larix gmelinii* the stage of mass mortalities; D, broadleaf *Pinus koraiensis* forests.

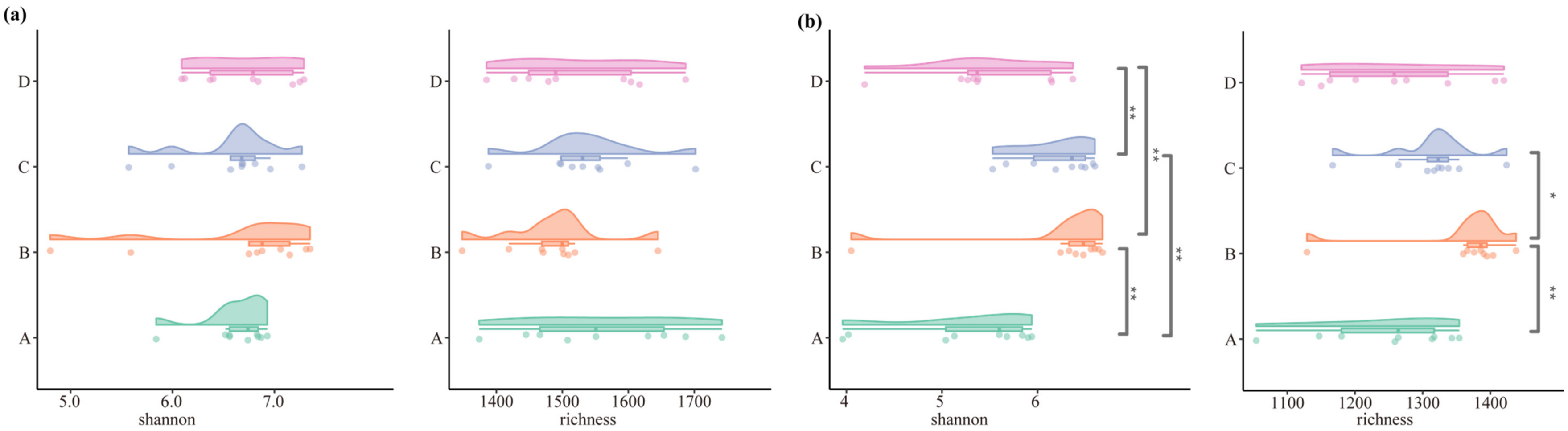

Figure S2. Fungal diversity during natural regeneration stages in different stands, (a) upper soil (0-20 cm), (b) lower soil (20-40 cm). A, *Larix gmelinii* pure forest; B, *Juglans mandshurica* at the stage of budding; C, *Larix gmelinii* the stage of mass mortalities; D, broadleaf *Pinus koraiensis*.

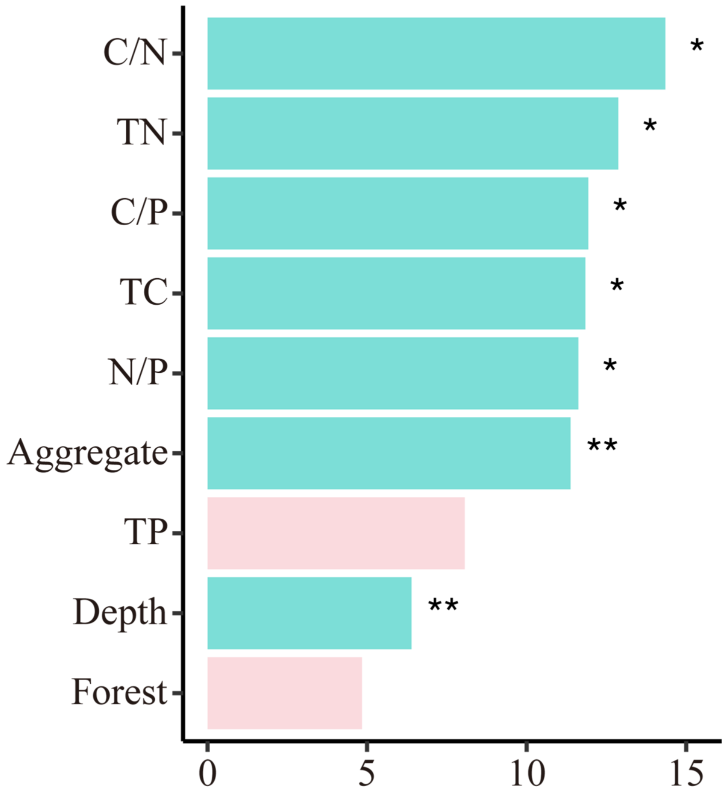

Figure S3. Random forest analysis of soil nutrient and fungal diversity within different agglomerate grain sizes during natural regeneration of forest stands. ns,p<0.05;*,p>0.05;**p>0.01.

Table S1. Nutrient and stoichiometric ratios within soil aggregate fractions at different stages of stand regeneration. Data are presented as Mean ± SEM. Lowercase letters represent the significance of differences between different aggregate fractions, and uppercase letters represent the significance of differences between stands ($p<0.05$). The different aggregate fractions are micro-aggregates (MI), small macro-aggregates (SM), and large macro-aggregates (LM). A, *Larix gmelinii* pure forest; B, *Juglans mandshurica* at the stage of budding; C, *Larix gmelinii* the stage of mass mortalities; D, broadleaf *Pinus koraiensis*.

	Aggregate	Topsoil				Subsoil			
		A	B	C	D	A	B	C	D
TC	MI	17.61±0.45Cb	27.59±0.31Ab	22.65±0.2Ba	16.44±0.08Da	4.09±0.08Ca	7.84±0.22Aa	4.28±0.01Ca	5.99±0.08Ba
	SM	19.38±0.69Ca	29.93±0.46Aa	19.74±1.01Bb	15.57±0.43Db	3.18±0.06Db	5.28±0.03Ac	3.63±0.03Cb	4.08±0.09Bc
	LM	15.77±0.16Cc	19.47±0.22Ac	18.45±0.79Bb	14.15±0.09Dc	3.15±0.04Cb	5.98±0.09Ab	3.01±0.05Dc	4.33±0.07Bb
TN	MI	1.37±0.03Ca	1.97±0.01Aa	1.76±0.02Ba	1.21±0.03Da	0.4±0.01Da	0.64±0.02Aa	0.44±0.02Ca	0.52±0.01Ba
	SM	1.33±0.04Ca	1.95±0.01Aa	1.47±0.06Bb	1.08±0.01Db	0.32±0.01Ab	0.39±0.02Ac	0.37±0.01ABb	0.36±0.01Bb
	LM	1.12±0.01Cb	1.45±0.02Ab	1.39±0.05Bb	1±0.01Dc	0.32±0.01Cb	0.44±0.01Ab	0.31±0.02Cc	0.35±0.02Bb
TP	MI	1.37±0.06Aa	1.18±0.04Ba	1.37±0.02Aa	1.23±0.02Ba	0.86±0.03Da	1.01±0.03Ba	1.09±0.06Aa	0.98±0.04Ba
	SM	1.34±0.07Aa	1.15±0.04Bab	1.18±0.08Bb	1.12±0.03Bb	0.78±0.02Bb	0.76±0.05Bb	0.94±0.02Ab	0.78±0.03Bb
	LM	1.36±0.02Aa	1.08±0.05Bb	1.3±0.05Aa	1.14±0.01Bb	0.71±0.02Cc	0.82±0.01Bb	0.9±0.05Ab	0.82±0.01Bb
C/N	MI	12.88±0.14Bc	14.03±0.12Ab	12.91±0.03Bb	13.65±0.36Ab	10.35±0.19Ca	12.2±0.01Ab	9.7±0.39Da	11.57±0.31Bab
	SM	14.61±0.1Ba	15.34±0.13Aa	13.4±0.21Ba	14.47±0.3Ca	9.88±0.12Cb	13.46±0.34Aa	9.72±0.19Ca	11.46±0.33Bb
	LM	14.08±0.29Ab	13.48±0.13Bc	13.26±0.07Ba	14.13±0.09Aab	9.8±0.2Cb	13.53±0.26Aa	9.84±0.4Ca	12.23±0.44Ba
N/P	MI	1±0.05Ca	1.67±0.05Aa	1.28±0.02Ba	0.98±0.02Ca	0.46±0.02Ca	0.64±0.01Ba	0.4±0.04Da	0.53±0.02Ba
	SM	0.99±0.08Ca	1.7±0.06Aa	1.26±0.12Ba	0.96±0.03Ca	0.41±0.01BCc	0.52±0.05Ab	0.4±0.01Cab	0.46±0.02Bb
	LM	0.82±0.01Cb	1.34±0.05Bb	1.07±0.04Cb	0.88±0Cb	0.46±0.02Ba	0.54±0.01Ab	0.34±0.03Cb	0.43±0.03Bb
C/P	MI	12.86±0.83Cab	23.38±0.61Ab	16.55±0.25Ba	13.32±0.31Ca	4.78±0.09Ca	7.79±0.16Aa	3.92±0.2Da	6.15±0.33Ba
	SM	14.54±1.29BCa	26.05±1.18Aa	16.85±1.65Ba	13.88±0.66Ca	4.11±0.06Cc	6.93±0.5Ab	3.87±0.09Ca	5.25±0.29Bb

LM	11.57±0.3Cb	17.99±0.65Ac	14.17±0.57Bb	12.38±0.11Cb	4.47±0.16Cb	7.29±0.1Aab	3.34±0.19Db	5.28±0.12Bb

Table S2. Two-way ANOVA analysis of soil nutrition. TC, total carbon; TN, total nitrogen; TP, total phosphorus; C/N, carbon to nitrogen ratio; C/P, carbon to phosphorus ratio; N/P, nitrogen to phosphorus ratio.

	Factor	0-20cm		20-40cm	
		f	p	f	p
TC	Aggregate	282.711	0	1116.68	0
	Forest	724.821	0	2067.01	0
	Aggregate*Forest	66.878	0	81.592	0
TN	Aggregate	400.397	0	466.565	0
	Forest	964.319	0	209.677	0
	Aggregate*Forest	43.38	0	36.5	0
TP	Aggregate	13.293	0	99.399	0
	Forest	46.05	0	52.346	0
	Aggregate*Forest	3.379	0.015	3.858	0.008
C/N	Aggregate	99.17	0	5.518	0.011
	Forest	55.18	0	258.318	0
	Aggregate*Forest	19.226	0	7.925	0
C/P	Aggregate	65.064	0	27.643	0
	Forest	256.216	0	438.274	0
	Aggregate*Forest	9.996	0	3.728	0.009
N/P	Aggregate	55.323	0	26.684	0
	Forest	264.576	0	80.442	0
	Aggregate*Forest	3.868	0.008	5.045	0.002

Table S3. Alpha-diversity of fungal communities. Lowercase letters indicate statistical differences ($p < 0.05$) between agglomerate fractions and uppercase letters indicate stage of stand renewal. MI, microaggregates; SM, small macroaggregates; LM, large macroaggregates. A, *Larix gmelinii* pure forest; B, *Juglans mandshurica* at the stage of budding; C, *Larix gmelinii* the stage of mass mortalities; D, broadleaf *Pinus koraiensis* forests.

Aggregate	Forest	Topsoil		Subsoil	
		shannon	richness	shannon	richness
A	A	6.76±0.17aA	1694.33±44.46aA	5.89±0.05aA	1324.67±15.95aA
	B	6.88±0.16abA	1480.67±18.5aB	6.4±0.16aA	1376.67±23.86aA
	C	6.82±0.14aA	1599±103aA	6.02±0.53aA	1335±81.5aA
	D	7.11±0.28aA	1628±51.39aA	6.22±0.13aA	1388±44.64aA
B	A	6.74±0.19aAB	1563±61.88bA	5.28±0.35abB	1292.33±53.46aB
	B	7.27±0.11aA	1476±49.57aA	6.57±0.08aA	1406.33±27.79aA
	C	6.27±0.61aB	1547.33±14.22aA	6.38±0.17aA	1329.67±23.54aAB
	D	6.55±0.57aAB	1476.33±123.62aA	4.89±0.6bB	1190.33±58.96bC
C	A	6.4±0.51aA	1428.33±48.21cA	4.53±0.93bA	1127±65.34bA
	B	5.76±1.05bA	1504±149.07aA	5.69±1.43aA	1298.33±146.81aA
	C	6.65±0.64aA	1467±68.94aA	6.21±0.48aA	1276±94.69aA
	D	6.45±0.36aA	1472.67±21.22aA	5.36±0.03bA	1199.33±77.51bA

Factor (Df)	f	p	f	p	f	p	f	p
Aggregates (2)	4.48	**0.02**	9.67	**0.00**	4.28	**0.03**	10.82	**0.00**
Forests (3)	0.10	0.96	1.58	0.22	6.84	**0.00**	5.04	**0.01**
Aggregates ´ Forests (6)	2.21	0.08	2.18	0.08	2.12	0.09	2.17	0.08

Table S4. Relative abundance (%) of fungal phyla (top 5). MI, microaggregates; SM, small macroaggregates; LM, large macroaggregates. A, *Larix gmelinii* pure forest; B, *Juglans mandshurica* at the stage of budding; C, *Larix gmelinii* the stage of mass mortalities; D, broadleaf *Pinus koraiensis* forests.

		Basidiomycota		Mortierellomycota		Ascomycota		unclassified_k_Fungi		others	
		Topsoil	Subsoil	Topsoil	Subsoil	Topsoil	Subsoil	Topsoil	Subsoil	Topsoil	Subsoil
MI	A	23.34±0.81	52.88±0.80	41.13±4.77	20.89±4.43	21.19±2.25	19.4±2.87	12.92±7.6	5.84±1.69	1.40±0.21	0.99±0.84
	B	14.06±0.45	44.07±3.31	49.02±1.42	25.86±2.34	26.42±0.99	24.24±4.53	8.98±0.33	5.25±0.90	1.52±0.21	0.57±0.04
	C	15.30±0.40	36.79±5.00	41.54±2.82	32.66±2.37	26.11±1.41	22.8±4.74	15.8±1.46	7.20±1.13	1.25±0.47	0.55±0.20
	D	18.11±3.17	37.91±3.54	26.91±7.04	28.83±3.72	39.76±4.08	24.67±1.48	13.68±1.48	7.63±0.89	1.54±0.17	0.96±0.10
SM	A	32.99±4.50	62.20±7.39	34.43±2.96	19.40±3.85	21.86±3.70	13.68±2.64	9.53±1.77	4.24±1.00	1.17±0.24	0.47±0.04
	B	20.21±1.41	42.33±5.37	31.75±2.41	22.72±4.26	36.97±1.53	28.99±2.66	10.1±0.51	5.33±0.68	0.95±0.02	0.61±0.07
	C	28.28±20.31	23.44±1.77	35.09±12.4	38.76±4.19	23.64±5.90	26.17±0.94	12.09±2.71	11.01±1.5	0.88±0.20	0.60±0.08
	D	31.11±13.31	57.04±7.19	16.00±4.11	17.85±2.55	39.62±13.45	17.89±3.30	12.26±4.00	6.60±1.37	1.01±0.21	0.61±0.16
LM	A	33.7±10.97	66.15±16.25	22.87±2.9	15.13±7.20	22.15±0.97	14.82±6.91	19.19±12.85	3.47±1.96	2.08±0.37	0.42±0.32
	B	9.26±4.04	50.15±19.4	15.61±9.91	19.52±7.40	69.25±14.51	21.42±9.05	5.43±3.14	8.03±6.46	0.44±0.33	0.87±0.48
	C	20.38±5.12	23.23±6.75	35.64±9.26	33.77±5.17	25.78±6.14	26.27±6.24	16.84±7.43	15.88±4.6	1.35±0.94	0.84±0.30
	D	24.34±5.30	52.33±2.46	18.13±12.96	21.62±4.79	39.56±15.64	17.85±4.32	15.8±4.88	7.47±2.63	2.16±0.88	0.72±0.19

Table S5. Relative abundance (%) of fungal genera (top 10). MI, microaggregates; SM, small macroaggregates; LM, large macroaggregates. A, *Larix gmelinii* pure forest; B, *Juglans mandshurica* at the stage of budding; C, *Larix gmelinii* the stage of mass mortalities; D, broadleaf *Pinus koraiensis* forests.

Depth	Aggregate	Forest	Mortierella	unclassified_k_Fungi	Entoloma	Sebacina	unclassified_p_Ascomycota	unclassified_o_Helotiales	Tomentella	Leptodontidium	unclassified_c_Leotiomycetes	Trechispora
0-20 cm	MI	A	40.87±4.73	12.92±7.6	3.19±0.26	2.78±0.07	1.56±0.25	2.64±0.27	3.70±0.19	0.19±0.03	1.67±0.09	0.33±0.12
		B	47.42±1.20	8.98±0.33	2.09±0.04	0.41±0.05	1.52±0.12	2.99±0.16	0.80±0.09	0.16±0.01	3.19±1.06	0.20±0.03
		C	41.27±2.80	15.8±1.46	1.66±0.08	0.36±0.01	2.35±0.13	3.55±0.36	0.69±0.06	0.28±0.26	1.86±0.42	0.23±0.06

		D	26.76±7.08	13.68±1.48	0.70±0.07	3.81±0.34	3.82±0.66	3.83±0.60	0.32±0.08	11.44±0.86	1.91±0.29	0.21±0.18
	SM	A	34.21±2.95	9.53±1.77	7.55±7.97	4.74±1.56	1.67±0.49	3.19±0.90	5.25±2.12	0.19±0.04	1.65±0.23	0.72±0.51
		B	30.88±2.41	10.10±0.51	2.1±0.32	0.37±0.09	2.86±0.47	6.44±1.94	0.73±0.29	0.20±0.04	2.20±1.09	0.33±0.31
		C	34.89±12.34	12.09±2.71	2.06±0.93	0.37±0.06	1.79±0.23	4.48±2.58	0.77±0.29	0.22±0.07	1.53±0.57	0.2±0.05
		D	15.90±4.15	12.26±4.01	1.25±1.04	6.61±2.26	4.91±2.73	4.20±1.35	0.44±0.17	11.56±1.60	1.8±0.99	0.14±0.08
	LM	A	22.69±2.87	19.19±12.85	2.01±0.35	9.76±5.15	1.08±0.23	2.63±0.23	7.07±3.20	0.17±0.04	1.89±0.54	0.31±0.08
		B	15.04±9.52	5.43±3.14	0.95±0.42	0.23±0.11	1.84±0.28	19.64±18.33	0.39±0.14	0.60±0.26	1.72±0.14	0.16±0.02
		C	35.43±9.28	16.84±7.43	1.62±0.32	0.39±0.02	2.9±0.96	3.09±0.31	0.75±0.18	0.32±0.20	1.49±0.38	0.19±0.04
		D	18.06±12.92	15.8±4.88	0.63±0.19	6.87±1.10	3.92±1.30	4.96±2.92	0.34±0.22	13.13±4.97	1.37±0.40	0.1±0.07
20-40 cm	MI	A	19.94±5.04	5.84±1.69	27.79±1.05	1.28±0.33	2.54±1.00	1.84±0.64	8.64±0.97	0.19±0.12	1.51±0.75	0.30±0.17
		B	25.36±2.37	5.25±0.90	13.31±2.04	0.34±0.02	3.43±0.73	2.73±0.75	7.50±1.59	0.29±0.05	4.45±1.60	0.20±0.02
		C	32.4±2.33	7.2±1.13	1.34±0.13	0.74±0.30	5.98±1.16	2.52±0.48	0.62±0.14	1.56±0.31	1.71±0.14	21.63±8.18
		D	28.67±3.71	7.63±0.89	3.01±0.30	20.35±3.27	4.10±0.62	2.12±0.17	0.49±0.13	6.75±0.18	1.33±0.51	0.09±0.02
	SM	A	19.17±3.88	4.24±1.00	35.05±4.59	1.08±0.10	2.32±0.49	1.65±0.65	7.06±1.13	0.13±0.13	1.67±0.61	0.23±0.07
		B	22.3±4.35	5.33±0.68	8.70±1.60	0.38±0.06	5.49±0.68	3.74±0.90	10.06±3.84	0.27±0.05	3.06±0.63	0.17±0.04
		C	38.48±4.13	11.01±1.5	1.18±0.42	0.65±0.27	7.85±1.92	2.56±0.15	0.46±0.19	1.71±0.9	1.55±0.63	7.87±1.37
		D	17.77±2.54	6.60±1.37	2.31±1.04	42.32±9.7	3.28±0.40	1.10±0.26	0.36±0.12	5.38±2.01	1.04±0.39	0.1±0.04
	LM	A	14.96±7.18	3.47±1.96	45.96±19.04	0.72±0.12	2.22±0.53	1.63±0.52	5.87±1.62	0.11±0.06	1.27±0.85	0.17±0.03
		B	18.99±6.81	8.03±6.46	26.17±28.79	0.45±0.03	6.21±2.91	2.42±1.56	7.40±4.21	0.15±0.09	2.90±1.85	0.19±0.03
		C	33.42±5.12	15.88±4.60	0.57±0.05	0.85±0.04	7.88±1.59	2.98±1.11	0.19±0.06	1.88±0.15	1.47±1.40	6.83±4.86
		D	21.52±4.83	7.47±2.63	7.06±3.05	29.4±3.06	3.27±0.70	1.07±0.29	0.77±0.79	4.98±2.69	1.49±1.02	0.06±0.01

Tables S6. Fungal covariance network topology of forest stand renewal processes and aggregate components. MI, microaggregates; SM, small macroaggregates; LM, large macroaggregates. A, *Larix gmelinii* pure forest; B, *Juglans mandshurica* at the stage of budding; C, *Larix gmelinii* the stage of mass mortalities; D, broadleaf *Pinus koraiensis* forests.

	A	B	C	D	LM	SM	MI
Node	41	50	37	32	64	61	54
Edge	314	345	186	196	274	200	183
Average degree	7.659	6.9	5.021	6.125	4.281	3.279	3.389
Network Diameter	4	5	3	3	5	5	5
Graph Density	0.191	0.141	0.14	0.198	0.068	0.055	0.064
Modularity	0.361	0.394	0.443	0.372	0.692	0.669	0.717
Average clustering coefficient	0.309	0.306	0.306	0.327	0.241	0.254	0.253
Average path length	1.558	1.694	1.456	1.503	1.739	1.902	1.617
Positive Proportion (%)	55.1	64.64	53.76	63.78	68.61	73.5	60.66
Negative Proportion (%)	44.9	35.36	43.24	36.22	31.39	26.5	39.34

Tables S7. Key taxa based on covariate network results for different stand natural regeneration stages. MI, microaggregates; SM, small macroaggregates; LM, large macroaggregates. A, *Larix gmelinii* pure forest; B, *Juglans mandshurica* at the stage of budding; C, *Larix gmelinii* the stage of mass mortalities; D, broadleaf *Pinus koraiensis* forests.

	label	kingdom	phylum	family	genus
	OTU_113	Fungi	Basidiomycota	Xenasmataceae	Xenasmatella
A	OTU_122	Fungi	Basidiomycota	Thelephoraceae	Tomentella
	OTU_123	Fungi	Chytridiomycota	Chytridiaceae	Dendrochytridium
	OTU_128	Fungi	unclassified_k_Fungi	unclassified_k_Fungi	unclassified_k_Fungi

	OTU_151	Fungi	Basidiomycota	Pluteaceae	Pluteus
	OTU_21	Fungi	unclassified_k_Fungi	unclassified_k_Fungi	unclassified_k_Fungi
	OTU_34	Fungi	Ascomycota	Pezizaceae	Peziza
	OTU_39	Fungi	Basidiomycota	Tricholomataceae	Bonomyces
	OTU_44	Fungi	Mortierellomycota	Mortierellaceae	Mortierella
	OTU_45	Fungi	Mortierellomycota	Mortierellaceae	Mortierella
	OTU_51	Fungi	unclassified_k_Fungi	unclassified_k_Fungi	unclassified_k_Fungi
	OTU_55	Fungi	Basidiomycota	Hymenogastraceae	unclassified_f_Hymenogastraceae
	OTU_65	Fungi	unclassified_k_Fungi	unclassified_k_Fungi	unclassified_k_Fungi
	OTU_6560	Fungi	unclassified_k_Fungi	unclassified_k_Fungi	unclassified_k_Fungi
	OTU_66	Fungi	Mortierellomycota	Mortierellaceae	Mortierella
	OTU_68	Fungi	Basidiomycota	Sebacinaceae	Sebacina
	OTU_78	Fungi	Basidiomycota	Ceratobasidiaceae	Ceratobasidium
	OTU_85	Fungi	Basidiomycota	unclassified_p_Basidiomycota	unclassified_p_Basidiomycota
	OTU_106	Fungi	Basidiomycota	Thelephoraceae	Tomentella
	OTU_120	Fungi	Ascomycota	Minutisphaeraceae	Minutisphaera
	OTU_12	Fungi	Ascomycota	Helotiales_fam_Incertae_sedis	Mycoarthris
	OTU_132	Fungi	Ascomycota	Helotiales_fam_Incertae_sedis	unclassified_f_Helotiales_fam_Incertae_sedis
B	OTU_134	Fungi	Basidiomycota	Psathyrellaceae	Psathyrella
	OTU_135	Fungi	Basidiomycota	Clavariaceae	Clavulinopsis
	OTU_13	Fungi	Basidiomycota	Piskurozymaceae	Solicoccozyma
	OTU_137	Fungi	Basidiomycota	unclassified_o_Agaricales	unclassified_o_Agaricales
	OTU_139	Fungi	unclassified_k_Fungi	unclassified_k_Fungi	unclassified_k_Fungi
	OTU_14	Fungi	Basidiomycota	Thelephoraceae	Tomentella

	OTU_15	Fungi	Ascomycota	unclassified_c_Leotiomycetes	unclassified_c_Leotiomycetes
	OTU_153	Fungi	Basidiomycota	Thelephoraceae	Tomentella
	OTU_179	Fungi	Ascomycota	Venturiaceae	Pseudoanungitea
	OTU_2354	Fungi	Mortierellomycota	Mortierellaceae	Mortierella
	OTU_242	Fungi	Ascomycota	Helotiales_fam_Incertae_sedis	Cadophora
	OTU_32	Fungi	Basidiomycota	unclassified_p_Basidiomycota	unclassified_p_Basidiomycota
	OTU_49	Fungi	Basidiomycota	unclassified_o_Russulales	unclassified_o_Russulales
	OTU_56	Fungi	Ascomycota	Sporormiaceae	Preussia
	OTU_58	Fungi	Ascomycota	Clavicipitaceae	Metapochonia
	OTU_63	Fungi	Ascomycota	Helotiales_fam_Incertae_sedis	unclassified_f_Helotiales_fam_Incertae_sedis
	OTU_64	Fungi	Mortierellomycota	Mortierellaceae	Mortierella
	OTU_71	Fungi	Ascomycota	Myxotrichaceae	Oidiodendron
	OTU_72	Fungi	Ascomycota	unclassified_o_Pleosporales	unclassified_o_Pleosporales
	OTU_79	Fungi	Basidiomycota	Cyphellaceae	unclassified_f_Cyphellaceae
	OTU_83	Fungi	Mortierellomycota	unclassified_p_Mortierellomycota	unclassified_p_Mortierellomycota
	OTU_88	Fungi	unclassified_k_Fungi	unclassified_k_Fungi	unclassified_k_Fungi
	OTU_8868	Fungi	Ascomycota	Helotiales_fam_Incertae_sedis	Mycoarthris
	OTU_107	Fungi	Ascomycota	unclassified_o_Helotiales	unclassified_o_Helotiales
	OTU_10	Fungi	Basidiomycota	Hydnodontaceae	Trechispora
	OTU_36	Fungi	Ascomycota	unclassified_p_Ascomycota	unclassified_p_Ascomycota
C	OTU_42	Fungi	unclassified_k_Fungi	unclassified_k_Fungi	unclassified_k_Fungi
	OTU_46	Fungi	Basidiomycota	Bolbitiaceae	Conocybe
	OTU_60	Fungi	Ascomycota	Helotiales_fam_Incertae_sedis	Cadophora
	OTU_62	Fungi	Ascomycota	Hypocreaceae	Trichoderma

	OTU_75	Fungi	Ascomycota	unclassified_o_Helotiales	unclassified_o_Helotiales
	OTU_86	Fungi	Ascomycota	unclassified_o_Pleosporales	unclassified_o_Pleosporales
	OTU_102	Fungi	Ascomycota	Cordycipitaceae	Beauveria
	OTU_1	Fungi	Basidiomycota	Sebacinaceae	Sebacina
	OTU_124	Fungi	Ascomycota	Helotiales_fam_Incertae_sedis	unclassified_f_Helotiales_fam_Incertae_sedis
	OTU_130	Fungi	Ascomycota	Pyronemataceae	Trichophaea
	OTU_143	Fungi	Ascomycota	unclassified_p_Ascomycota	unclassified_p_Ascomycota
	OTU_160	Fungi	Basidiomycota	Physalacriaceae	Armillaria
	OTU_18	Fungi	Basidiomycota	Clavulinaceae	Clavulina
	OTU_210	Fungi	Basidiomycota	unclassified_o_Russulales	unclassified_o_Russulales
D	OTU_38	Fungi	Basidiomycota	Agaricaceae	unclassified_f_Agaricaceae
	OTU_47	Fungi	Basidiomycota	Sebacinaceae	Sebacina
	OTU_59	Fungi	Basidiomycota	Entolomataceae	Entoloma
	OTU_70	Fungi	Ascomycota	Clavicipitaceae	unclassified_f_Clavicipitaceae
	OTU_7756	Fungi	unclassified_k_Fungi	unclassified_k_Fungi	unclassified_k_Fungi
	OTU_77	Fungi	Ascomycota	Herpotrichiellaceae	Exophiala
	OTU_7804	Fungi	unclassified_k_Fungi	unclassified_k_Fungi	unclassified_k_Fungi
	OTU_82	Fungi	Ascomycota	unclassified_o_Xylariales	unclassified_o_Xylariales
	OTU_94	Fungi	Ascomycota	unclassified_o_Thelebolales	unclassified_o_Thelebolales
	OTU_99	Fungi	Ascomycota	unclassified_p_Ascomycota	unclassified_p_Ascomycota

Table S8. Key taxa for different agglomerate fractions based on covariance network results. MI, microaggregates; SM, small macroaggregates; LM, large macroaggregates. A, *Larix gmelinii* pure forest; B, *Juglans mandshurica* at the stage of budding; C, *Larix gmelinii* the stage of mass mortalities; D, broadleaf *Pinus koraiensis* forests.

	label	kingdom	phylum	family	genus
LM	OTU_58	Fungi	Ascomycota	Clavicipitaceae	Metapochonia
	OTU_60	Fungi	Ascomycota	Helotiales_fam_Incertae_sedis	Cadophora
	OTU_67	Fungi	Ascomycota	unclassified_c_Leotiomycetes	unclassified_c_Leotiomycetes
	OTU_8868	Fungi	Ascomycota	Helotiales_fam_Incertae_sedis	Mycoarthris
	OTU_122	Fungi	Basidiomycota	Thelephoraceae	Tomentella
	OTU_151	Fungi	Basidiomycota	Pluteaceae	Pluteus
	OTU_47	Fungi	Basidiomycota	Sebacinaceae	Sebacina
	OTU_54	Fungi	Basidiomycota	Leucosporidiaceae	unclassified_f_Leucosporidiaceae
	OTU_68	Fungi	Basidiomycota	Sebacinaceae	Sebacina
	OTU_78	Fungi	Basidiomycota	Ceratobasidiaceae	Ceratobasidium
	OTU_91	Fungi	Basidiomycota	Podoscyphaceae	Cotylidia
	OTU_17	Fungi	Mortierellomycota	Mortierellaceae	Mortierella
	OTU_4	Fungi	Mortierellomycota	Mortierellaceae	Mortierella
	OTU_105	Fungi	unclassified_k_Fungi	unclassified_k_Fungi	unclassified_k_Fungi
	OTU_25	Fungi	unclassified_k_Fungi	unclassified_k_Fungi	unclassified_k_Fungi
	OTU_6560	Fungi	unclassified_k_Fungi	unclassified_k_Fungi	unclassified_k_Fungi
	OTU_88	Fungi	unclassified_k_Fungi	unclassified_k_Fungi	unclassified_k_Fungi
SM	OTU_179	Fungi	Ascomycota	Venturiaceae	Pseudoanungitea
	OTU_242	Fungi	Ascomycota	Helotiales_fam_Incertae_sedis	Cadophora
	OTU_41	Fungi	Ascomycota	Leotiaceae	Neobulgaria

	OTU_63	Fungi	Ascomycota	Helotiales_fam_Incertae_sedis	unclassified_f_Helotiales_fam_Incertae_sedis
	OTU_72	Fungi	Ascomycota	unclassified_o_Pleosporales	unclassified_o_Pleosporales
	OTU_99	Fungi	Ascomycota	unclassified_p_Ascomycota	unclassified_p_Ascomycota
	OTU_113	Fungi	Basidiomycota	Xenasmataceae	Xenasmatella
	OTU_129	Fungi	Basidiomycota	Agaricaceae	unclassified_f_Agaricaceae
	OTU_137	Fungi	Basidiomycota	unclassified_o_Agaricales	unclassified_o_Agaricales
	OTU_153	Fungi	Basidiomycota	Thelephoraceae	Tomentella
	OTU_155	Fungi	Basidiomycota	Typhulaceae	Typhula
	OTU_38	Fungi	Basidiomycota	Agaricaceae	unclassified_f_Agaricaceae
	OTU_53	Fungi	Basidiomycota	Ganodermataceae	Ganoderma
	OTU_79	Fungi	Basidiomycota	Cyphellaceae	unclassified_f_Cyphellaceae
	OTU_85	Fungi	Basidiomycota	unclassified_p_Basidiomycota	unclassified_p_Basidiomycota
	OTU_102	Fungi	Ascomycota	Cordycipitaceae	Beauveria
	OTU_70	Fungi	Ascomycota	Clavicipitaceae	unclassified_f_Clavicipitaceae
	OTU_86	Fungi	Ascomycota	unclassified_o_Pleosporales	unclassified_o_Pleosporales
MI	OTU_64	Fungi	Mortierellomycota	Mortierellaceae	Mortierella
	OTU_6580	Fungi	Mortierellomycota	Mortierellaceae	Mortierella
	OTU_66	Fungi	Mortierellomycota	Mortierellaceae	Mortierella
	OTU_83	Fungi	Mortierellomycota	unclassified_p_Mortierellomycota	unclassified_p_Mortierellomycota
	OTU_65	Fungi	unclassified_k_Fungi	unclassified_k_Fungi	unclassified_k_Fungi
